# Feasibility of a Heterogeneous Nanoscale Zero-Valent Iron Fenton-like Process for the Removal of Glyphosate from Water

**DOI:** 10.3390/molecules28052214

**Published:** 2023-02-27

**Authors:** Naveed Ahmed, Davide Vione, Luca Rivoira, Michele Castiglioni, Mihail S. Beldean-Galea, Maria Concetta Bruzzoniti

**Affiliations:** 1 Department of Chemistry, University of Turin, Via Pietro Giuria 5, 10125 Turin, Italy; 2 Faculty of Environmental Science and Engineering, Babes-Bolyai University, 400347 Cluj-Napoca, Romania

**Keywords:** glyphosate, ZVI, degradation, tap water, pH, Fenton, adsorption, heterogeneous reactions

## Abstract

Glyphosate is a widely used herbicide, and it is an important environmental pollutant that can have adverse effects on human health. Therefore, remediation and reclamation of contaminated streams and aqueous environments polluted by glyphosate is currently a worldwide priority. Here, we show that the heterogeneous nZVI–Fenton process (nZVI + H_2_O_2_; nZVI: nanoscale zero-valent iron) can achieve the effective removal of glyphosate under different operational conditions. Removal of glyphosate can also take place in the presence of excess nZVI, without H_2_O_2_, but the high amount of nZVI needed to remove glyphosate from water matrices on its own would make the process very costly. Glyphosate removal via nZVI–-Fenton was investigated in the pH range of 3–6, with different H_2_O_2_ concentrations and nZVI loadings. We observed significant removal of glyphosate at pH values of 3 and 4; however, due to a loss in efficiency of Fenton systems with increasing pH values, glyphosate removal was no longer effective at pH values of 5 or 6. Glyphosate removal also occurred at pH values of 3 and 4 in tap water, despite the occurrence of several potentially interfering inorganic ions. Relatively low reagent costs, a limited increase in water conductivity (mostly due to pH adjustments before and after treatment), and low iron leaching make nZVI–Fenton treatment at pH 4 a promising technique for eliminating glyphosate from environmental aqueous matrices.

## 1. Introduction

Glyphosate (GLY: N-(phosphonomethyl)glycine) is the world’s most-used herbicide, and it is employed to control weeds in both agricultural (herbaceous crops and trees) and non-agricultural areas [1]. GLY is so widely used owing to its broad spectrum (effectively killing any plant, with the exception of some genetically modified ones), high efficacy, good toxicological profile, and low cost [2,3]. Since its first commercialization in the 1970s [4], the use of GLY has steadily increased, with almost 600–700 thousand tons used annually, and an expected 740–920 thousand tons to be used by 2025 [5]. In the meanwhile, many harmful effects of GLY on plants, humans, and animals have been discovered, including the weakening of plant systems, endocrine disorders, and the disruption of animal metabolism [6,7,8]. GLY is potentially carcinogenic to humans, even if present at trace levels (<0.02 mg/L) in the environment [9]. Up to now, about 750 GLY-containing products have been used in 130 countries [1,10], but several countries have banned its use. The EU regulations have set a maximum permissible limit of 0.1 μg/L for GLY in water [11], but in some areas, its concentration is as high as 0.7 mg/L [12]. Although relatively low GLY concentrations have been detected in surface waters in Germany, Hungary, Switzerland, and northeastern Spain (0.1–2.5 μg/L, which is still often above the EU limit), high levels (up to 165 μg/L) have often been found in Denmark and France [8]. Eventually, an efficient strategy will be required to treat wastewaters and other streams that are contaminated with GLY before concentration of this compound reaches alarming levels and GLY diffuses into other compartments, such as the food chain.

Conventional water treatment methods, including adsorption, biological oxidation, and chemical oxidation, have all been used to remove GLY from water [13,14,15,16,17,18]. In recent decades, advanced oxidation processes (AOPs) have attracted much attention as promising methods for the abatement of organic pollutants [16]. The AOPs include numerous techniques based on the in situ formation of strong oxidants. Among those species, the hydroxyl radical (HO^•^) plays a central role in AOPs due to its high standard reduction potential (2.8 V vs. NHE), especially in acidic media. Being highly reactive and nonselective, HO^•^ can oxidize several organic compounds [19]. Main AOPs include ultraviolet (UV) irradiation, H_2_O_2_/UV, photocatalysis, ozonation, electrochemical oxidation, Fenton processes, and Fenton-like processes. Many of these have been used to remove GLY from wastewater [20,21,22,23,24,25].

Unfortunately, the majority of AOPs are too costly to be applied to the treatment of environmental water matrices, which are much more likely than wastewater to be contaminated by GLY. Oxidation with Fenton’s reagent is an AOP based on ferrous ions reacting with hydrogen peroxide to produce hydroxyl radicals and/or other oxidizing species (e.g., ferryl). Fenton is an effective and proven method for the abatement of several hazardous organic pollutants [16,22]. Over the past few years, heterogeneous Fenton-like processes using nanoscale zero-valent iron (nZVI) have attracted much attention due to their ability to efficiently remove heavy metals and organic contaminants [26,27,28,29,30,31,32,33]. Under acidic conditions, slowly released Fe^2+^ (or Fe^II^ occurring on the surface of nZVI) activates H_2_O_2_ to promote the production of HO^•^ (and/or ferryl), as described in reactions (1–2) [34,35]. The most important advantage of using nZVI, rather than conventional ferrous ions, is the rapid recycling of Fe^3+^ into Fe^2+^ at the surface of the nZVI, which can proceed as shown in reaction (3) [35]. Moreover, magnetic nZVI can be easily separated from the aqueous phase at the end of treatment.

Fe^0^ + H_2_O_2_ + 2 H^+^ → Fe^2+^ + 2 H_2_O (1)

Fe^2+^ + H_2_O_2_ → Fe^3+^ + OH^−^ + HO^•^ (2)

2 Fe^3+^ + Fe^0^ → 3 Fe^2+^ (3)

It has been reported that, by using nZVI (22.4 mg/L) and H_2_O_2_ (0.1 mmol/L) at pH 3, chlorpheniramine (10 mg/L) can be completely removed at room temperature [36]. Moreover, the efficiency of the degradation of polyvinyl alcohol (PVA) reached 94% in 1 min at pH 3 with a nZVI dosage of 5 mg/L and a H_2_O_2_ concentration of 1 × 10^−4^ mol/L [34]. Many approaches have been attempted to remove different contaminants from water using Fenton-related processes [37,38,39,40,41,42,43,44,45]. However, GLY removal from water using nZVI, through either adsorption or degradation (nZVI/H_2_O_2_ Fenton-like system), has not yet been reported.

This is the first work, to our knowledge, in which GLY removal was investigated by using nZVI + H_2_O_2_ at different pH values (a heterogeneous Fenton-like process). The removal efficiency was here maximized by optimizing the concentrations/loadings of both nZVI and H_2_O_2_. The comparative removal of GLY via either nZVI alone or nZVI + H_2_O_2_ was also studied so as to highlight the role of the nZVI–Fenton reaction in the process. Finally, nZVI–Fenton degradation of GLY was also investigated using tap water.

## 2. Results and Discussion

**Figure 1:**
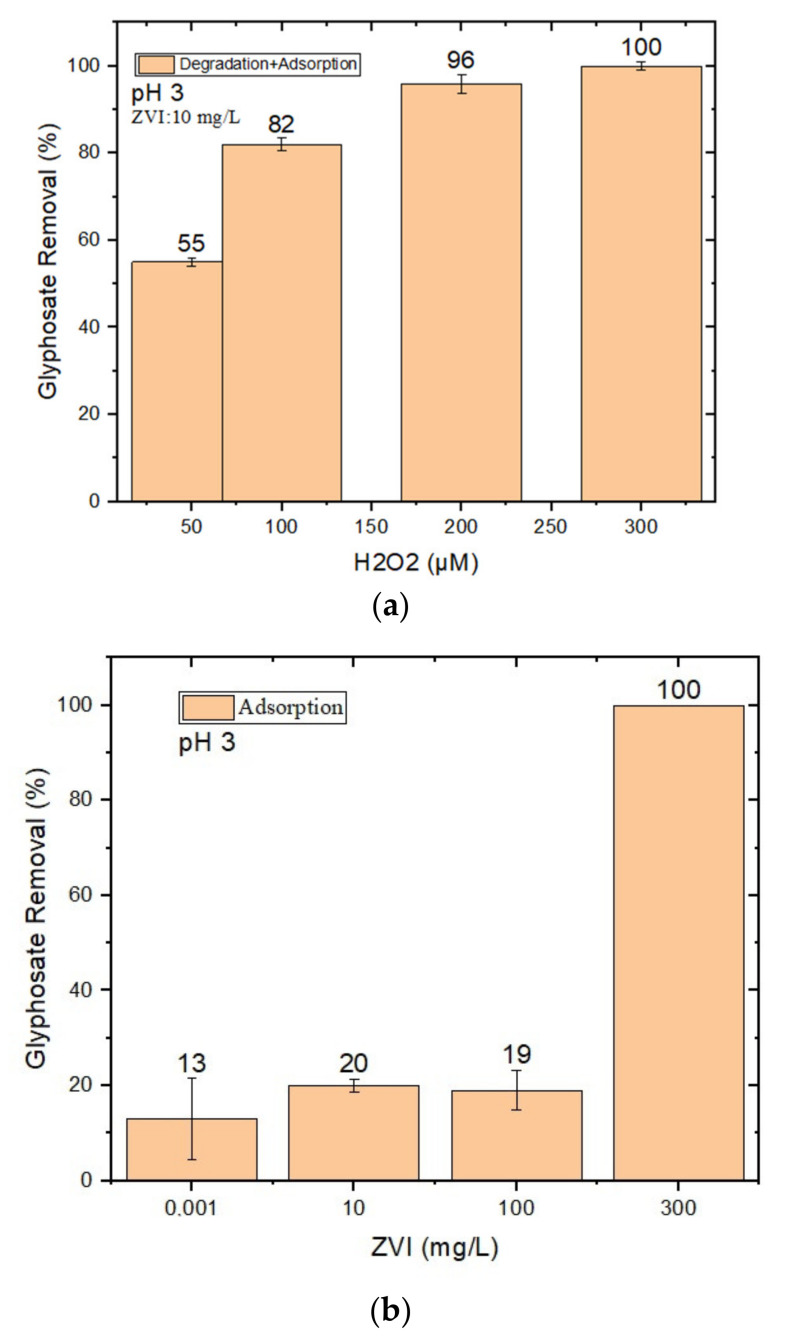
(**a**) Effect of H_2_O_2_ concentration (50–300 μM) on GLY removal via nZVI–Fenton at pH 3 at 10 mg/L nZVI loading, optimized to minimize adsorption; (**b**) GLY removal using different loadings of nZVI alone, without H_2_O_2_, at pH 3. The error bounds represent the standard deviation of triplicate experiments.

To optimize the concentrations/loadings of nZVI and H_2_O_2_, a number of experiments were performed at different pH values to investigate the efficiency of GLY degradation. Preliminary experiments with nZVI + H_2_O_2_ at pH 3 were carried out to highlight the Fenton degradation (Figure 1).

The loading of nZVI (varying in the range of 0.001–300 mg/L) was optimized while minimizing GLY removal by nZVI alone (which could involve adsorption, at least in part; see Figure 1b). Indeed, although complete GLY removal could be achieved by using 300 mg/L nZVI without H_2_O_2_ (Figure 1b), such a high nZVI loading would be impractical in water treatment applications due to the elevated costs of chemical reagents. (A quantity of 300 mg/L nZVI would cost approximately USD 0.24/m^3^ [46]).

Note that adsorption is not the only phenomenon that could account for the removal of GLY by nZVI alone, as shown in Figure 1b. Indeed, the nZVI–Fenton process can be activated by O_2_ alone, without H_2_O_2_, with the generation of both Fenton reactants [47]:

Fe^0^ + O_2_ + 2H^+^ → Fe^2+^ + H_2_O_2_ (4)

The minimum tested loading of nZVI (0.001 mg/L) was effective at minimizing adsorption, but it did not induce effective GLY degradation in the presence of H_2_O_2_. Moreover, such a low level of loading could not be obtained by direct weighting, but rather by the dilution of a stock nZVI suspension. Presumably due to the difficulty of fully homogenizing the stock suspension, the system lacked reproducibility in results.

Therefore, compromise loadings (10 mg/L, i.e., the lowest loading that could be achieved via direct weighting, or 20 mg/L) were chosen for the nZVI in subsequent experiments.

### 2.1. GLY Removal at pH 3

As shown in Figure 1a, complete GLY removal could be achieved at pH 3 by using 10 mg/L nZVI and 300 μM H_2_O_2_. Lower H_2_O_2_ concentrations were less effective, most likely because of lower hydroxyl radical (HO^•^) generation when H_2_O_2_ is low. Higher H_2_O_2_ concentrations were not tested because their use would increase treatment costs (300 μM H_2_O_2_ would cost approximately USD 0.009/m^3^ [46]) and because effectiveness would decrease at a certain point due to HO^•^ scavenging by the H_2_O_2_ itself [48]:

H_2_O_2_ + HO^•^ → HO_2_^•^ + H_2_O (5)

### 2.2. GLY Removal at pH 4

**Figure 2:**
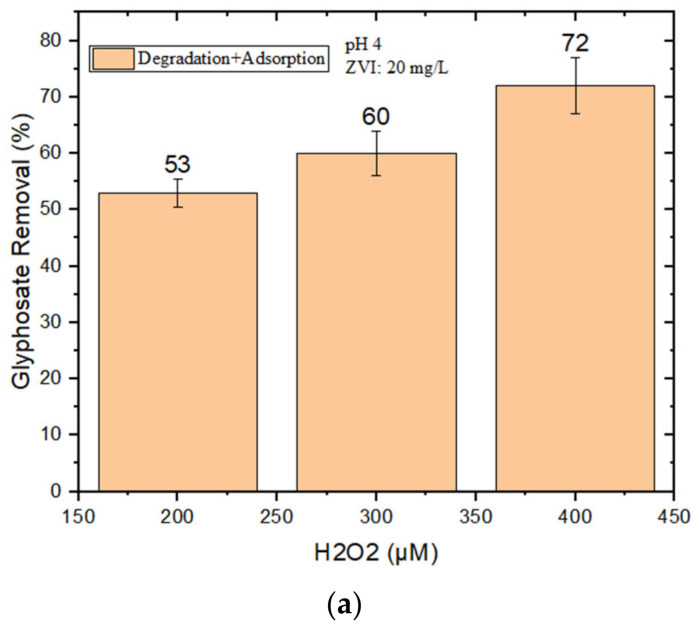

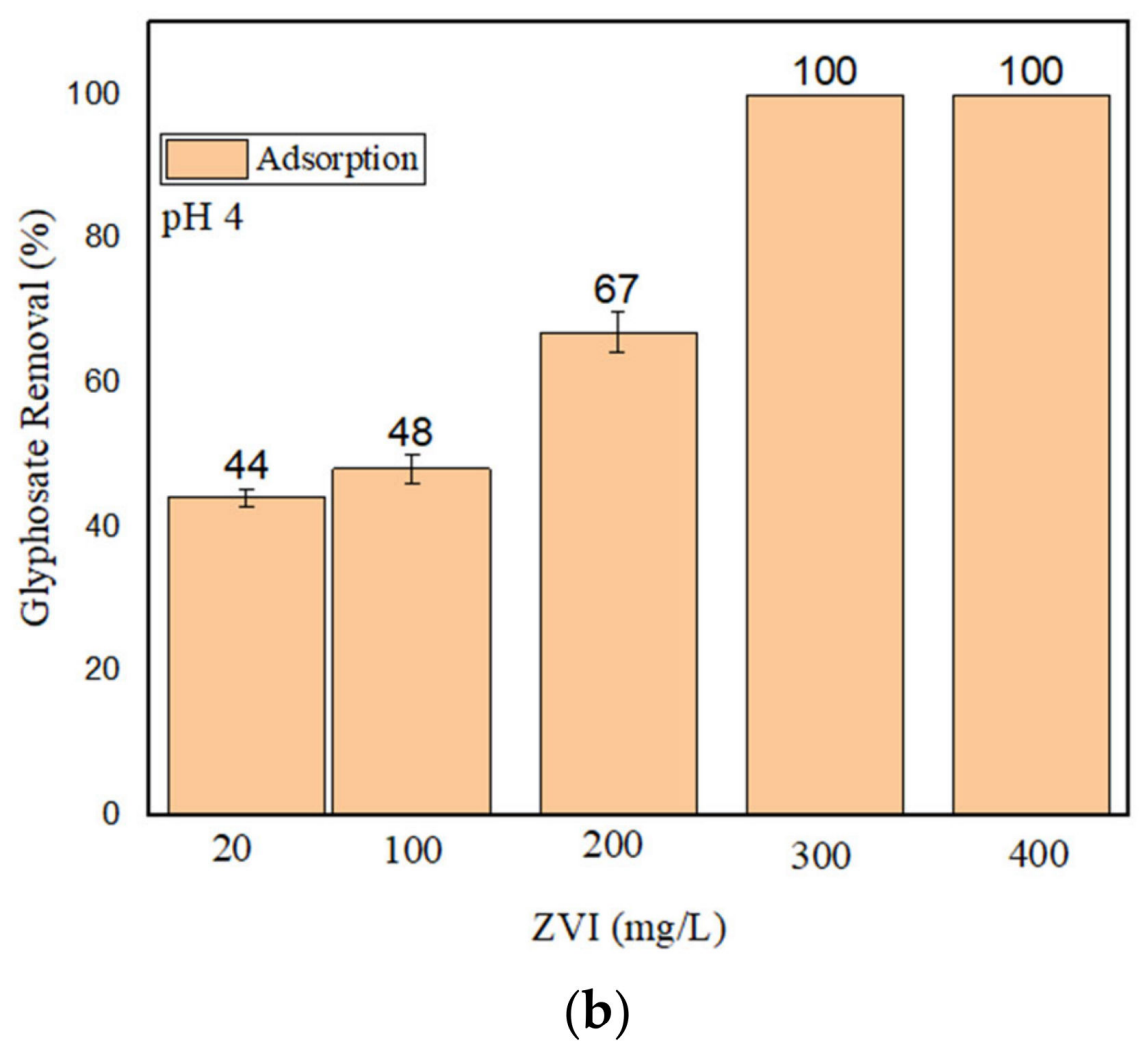
(**a**) GLY removal (%) by nZVI (20 mg/L) and H_2_O_2_ (200–400 μM) at pH 4; (**b**) GLY removal at pH 4 by nZVI alone (with different loadings).

It was not possible to achieve satisfactory GLY removal at pH 4 in the presence of 10 mg/L nZVI + H_2_O_2_. For this reason, higher loadings of nZVI (20–40 mg/L) were tested, but the adsorption of GLY by nZVI alone (i.e., without H_2_O_2_) was also checked. Limited adsorption was observed with 20 mg/L nZVI, which was then used to perform further degradation experiments at different concentrations of H_2_O_2_ (200–400 μM). However, as shown in Figure 2a, the maximum GLY removal by nZVI + H_2_O_2_ was, at most, only 72% when using 400 μM H_2_O_2_ at pH 4.

**Figure 3:**
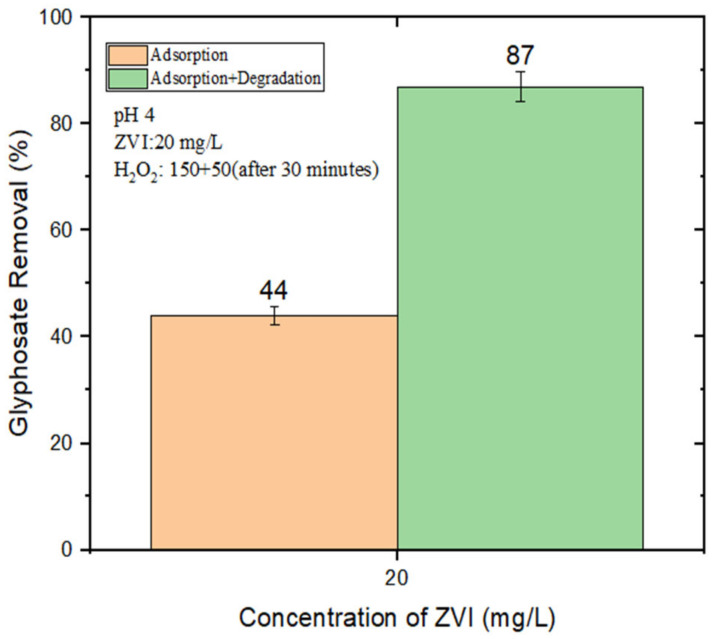
GLY removal (%) by nZVI (20 mg/L) and, where applicable (“Adsorption + Degradation”), H_2_O_2_ (150 μM at t = 0 + 50 μM at t = 30 min) at pH 4.

An alternative strategy, proposed by Minella et al. (2019), consists in the stepwise addition of H_2_O_2_. This procedure allows for the use of a relatively high overall amount of H_2_O_2_ (thereby providing a sufficiently high total amount of generated HO^•^), while simultaneously minimizing HO^•^ scavenging by the H_2_O_2_ itself. The rationale is that the H_2_O_2_ becomes progressively degraded as the reaction goes on and, if H_2_O_2_ is added stepwise, it never reaches excessive concentration values at any time point [31]. By adding 150 μM of H_2_O_2_ at the beginning, plus an additional 50 μM at 30 min after the start of the reaction, we obtained 87% GLY removal with 20 mg/L nZVI at pH 4 (Figure 3).

### 2.3. GLY Removal at pH Values of 5 and 6

Several combinations of nZVI loadings (10–30 mg/L) and H_2_O_2_ concentrations, including multiple additions of different amounts of the latter, were attempted for the removal of GLY at pH values of 5 and 6 via nZVI–Fenton. However, no GLY degradation at all was observed. In particular, the tested conditions at both pH 5 and 6 were as follows: (i) 10 mg/L nZVI, 150 μM H_2_O_2_ at the beginning of the reaction, plus an additional 50 μM H_2_O_2_ at 30 min reaction time; (ii) 20 mg/L nZVI, 200 μM H_2_O_2_ at the beginning of the reaction, plus an additional 50 μM H_2_O_2_ at 30 min reaction time; (iii) 30 mg/L nZVI, 300 μM H_2_O_2_ at the beginning of the reaction, plus an additional 50 μM H_2_O_2_ at 30 min reaction time.

The most likely explanation for the lack of GLY degradation at pH values of 5 and 6 is the decrease in efficiency of the Fenton reaction with the increase in the pH value (and of the nZVI–Fenton reaction as well [31,46]). Actually, a major issue in the Fenton process is Fe(III) recycling to Fe(II) [46,49]. As the pH increases, the decreasing solubility of the Fe(III) hampers its reduction to Fe(II), slowing down the Fenton degradation [49].

### 2.4. GLY Removal from Tap Water

GLY removal was then studied using GLY-spiked tap water at pH values of 3 and 4 (the pH was adjusted by H_2_SO_4_; the original tap water pH was 7.6–7.7), starting with the loadings/concentrations of nZVI and H_2_O_2_ already optimized in ultra-pure water. At pH 3, with 10 mg/L nZVI and 300 μM H_2_O_2_, the GLY removal efficiency reached 80%. Unfortunately, it was not possible to improve the removal percentage, even by employing the stepwise addition method. In particular, the achieved removal percentages at 1 h were as follows: (i) 54% with 10 mg/L nZVI, 150 μM H_2_O_2_ at the beginning of the reaction, plus an additional 50 μM H_2_O_2_ at 30 min reaction time; (ii) 72% with 10 mg/L nZVI, 200 μM H_2_O_2_ at the beginning of the reaction, plus an additional 50 μM H_2_O_2_ at 30 min reaction time; (iii) 75% with 10 mg/L nZVI, 350 μM H_2_O_2_ at the beginning of the reaction, plus an additional 50 μM H_2_O_2_ at 30 min reaction time; (iv) 76% with 10 mg/L nZVI, 400 μM H_2_O_2_ at the beginning of the reaction, plus an additional 50 μM H_2_O_2_ at 30 min reaction time.

The lower level of GLY removal from tap water, compared to ultra-pure water, might be tentatively ascribed to the presence of common inorganic anions in tap water (Cl^−^, SO_4_^2−^, and NO_3_^−^) (see Table 1). Some anions, and especially chloride, are able to scavenge HO^•^ at pH 3 to produce different reactive transient species that are, however, significantly less reactive than HO^•^ itself [48], thereby lowering the nZVI–Fenton efficiency [33]. Furthermore, the scavenging of HO^•^ by H_2_O_2_ would become comparatively less important in tap water that already contains other HO^•^ scavengers, thereby decreasing the effectiveness of the stepwise H_2_O_2_ addition method.

**Figure 4:**
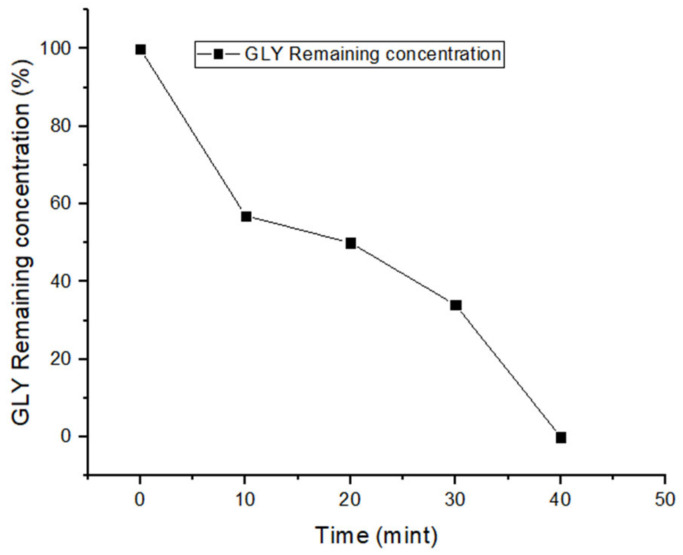
Kinetics of GLY removal from tap water at pH 4.

Very interestingly, it was possible to achieve 100% GLY removal from tap water at pH 4 by using 20 mg/L nZVI, 150 μM of H_2_O_2_ at the beginning of the reaction, plus an additional 50 μM at 30 min reaction time. Complete removal of GLY was achieved in 40 min, and degradation at 30 min was around 66% (Figure 4). A possible reason for the better performance of nZVI–Fenton at pH 4, as compared to pH 3, is that the reaction rate between chloride and HO^•^ decreases with increases in the pH value, because the HO^•^ + Cl^−^ reaction step becomes partially reversible [48].

The pH and conductivity values of the GLY-spiked tap water were monitored during the nZVI–Fenton treatment. In the case of the experiments carried out at pH 3, the pH value was quite stable and ranged between 3–3.2. Initial water conductivity was 0.53–0.58 mS/cm; it increased to around 0.9–1.1 mS/cm upon acidification to pH 3, and then it remained reasonably stable during the advancement of the reaction.

As far as the degradation experiments at a pH value of 4 are concerned, initial tap-water conductivity was around 0.5–0.55 mS/cm, and it increased to around 0.6 mS/cm upon acidification to pH 4. A further increase, by ~0.1 mS/cm units, is expected to take place in the final neutralization step, intended to bring the pH to at least 6 to allow for discharge in the environment. Despite the increase in conductivity, it would still be possible to reuse treated water in several fields, including some of the most demanding in terms of water conductivity (e.g., agriculture [50]). The pH value was reasonably stable during the reaction (4.0–4.3). Leached iron at pH 4 was quantified as well; the concentration of dissolved Fe was undetectable before treatment and amounted to 2.5 mg/L at the end of the treatment. This means that around 12% of the initially added nZVI (20 mg/L) underwent dissolution at pH 4. When compared with Fe wastewater limits (4 mg/L according to Italian law), a dissolved Fe concentration of 2.5 mg/L suggests that treated water can be safely discharged without the need for additional steps of iron removal (apart from the easy magnetic separation of nZVI).

Under optimal conditions in tap water (pH 4, 20 mg/L nZVI, 150 + 50 μM H_2_O_2_), the GLY removal capacity of nZVI–Fenton can be calculated as at least 100 mg _GLY_/g _nZVI_. This value exceeds the capacity of adsorption systems based on metals with high complexation capabilities towards P-containing ligands [51] and, to an even higher extent, the adsorption capacity of activated carbon [52], which is a widespread adsorbent used in tertiary treatments at most potabilization facilities. Therefore, the studied Fenton-like process appears to make better use of the solid reagent, as compared to adsorptive techniques.

### 2.5. Cost Estimates for GLY Removal from Tap Water

Based on the results reported in the previous section, a preliminary and partial estimate of the costs of the nZVI–Fenton process can be carried out within the framework of expenditures for chemical reagents per m^3^ of treated water. This estimate is significant, in that reagents are a major expenditure in Fenton treatments [53]. The necessary infrastructure, including basins for water treatment, also contributes to the treatment costs, but in the case of Fenton techniques, the required infrastructure is much simpler and considerably less costly, as compared to ozonation, for example. [53].

The costs of bulk chemicals that were used as reference for calculations are as follows [46]: USD 800/ton for nZVI, USD 500/ton for H_2_O_2_, USD 240/ton for H_2_SO_4_, and USD 120/ton for CaO. Note that CaO is a relatively cheap base that can be used to adjust pH after treatment. Indeed, because the environmental discharge of treated water with a pH value of 3 or 4 is not permitted, the water’s pH should be increased to at least 6 in order to avoid damaging the receiving bodies of water [50].

**Figure 5:**
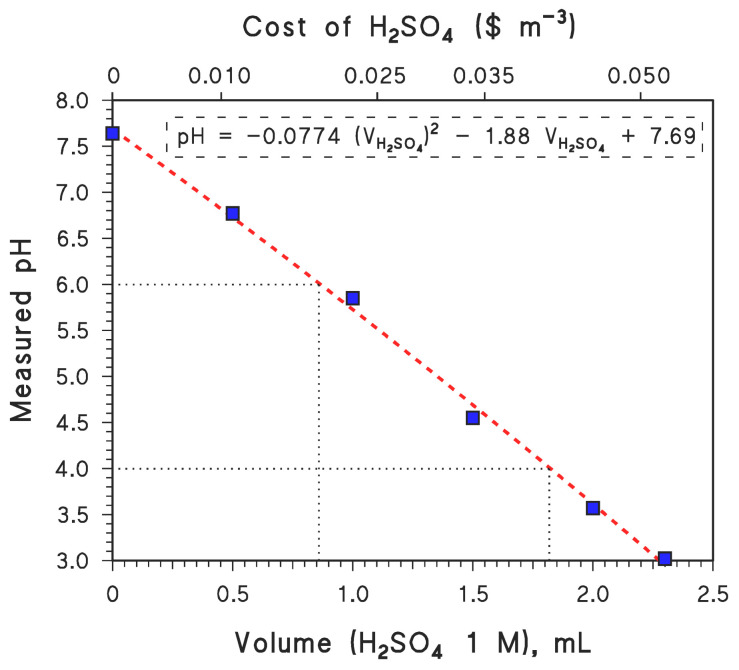
Results of tap-water titration with H_2_SO_4_ (measured pH, as a function of added volume of H_2_SO_4_ 1 M). The upper *X*-axis plots the cost of H_2_SO_4_ addition, based on an estimate of USD 240 (ton H_2_SO_4_)^−1^ [46]. The reported phenomenological equation is a second-order polynomial fit. The volumes of H_2_SO_4_ needed to bring the pH to values of 4 and 6 are highlighted. (The latter allows for the calculation of the amount of CaO needed to fix the pH at 6 at the end of the treatment.).

In the case of GLY degradation at pH 4, with 20 mg/L nZVI and 200 μM H_2_O_2_ (i.e., 150 μM + 50 μM), cost estimates are USD 0.016/m^3^ for nZVI and USD 0.006/m^3^ for H_2_O_2_. The cost of H_2_SO_4_ can be estimated by carrying out tap-water titration and then measuring the amount of H_2_SO_4_ that is required to adjust the water’s pH to any given value (pH 4 in the present case; see Figure 5). On this basis, one can conclude that the adjustment of the pH value to 4 would require 1.8 mol H_2_SO_4_ m^−3^, which translates to a cost of USD 0.041/m^3^ for H_2_SO_4_ [46]. Moreover, again based on water titration data, one would also need 1 mol CaO m^−3^ to finally adjust the pH value to at least 6 for eventual discharge or reuse, which entails an added cost of USD 0.0067/m^3^ [46]. Overall, the elimination of GLY from water using the nZVI–Fenton technique at a pH of 4 would cost USD 0.070/m^3^ in chemical reagents.

Treatment at pH 3 would cost USD 0.008/m^3^ for the nZVI, USD 0.009/m^3^ for the H_2_O_2_, USD 0.052/m^3^ for the H_2_SO_4_, and USD 0.010/m^3^ for the CaO, for a total of USD 0.079/m^3^ (i.e., a slightly higher cost, as compared to pH 4, to achieve a lower level of GLY removal). Moreover, higher conductivity increases (up to 1–1.5 mS/cm) were observed upon water treatment at pH 3, as compared to at pH 4, and 8 mg/L iron leached from 10 mg/L nZVI at pH 3 (implying 80% dissolution of initially occurring nZVI). This means that a further step of dissolved-iron removal before discharge would be required for treatment at pH 3.

By comparison, the cost of traditional wastewater treatment (including sedimentation and activated sludge, which is often unable to remove GLY) is in the range of USD 0.3–0.4/m^3^ [31,46]. Ozonation costs would be in the same range (about 4 times as much as the cost of chemicals in Fenton [53]), which makes nZVI–Fenton a potentially very competitive and effective technique for the removal of GLY from aqueous streams.

## 3. Experimental

### 3.1. Reagents and Materials

All chemical reagents used in the study were of analytical grade and were used without further purification. Nanoscale zero-valent iron (nZVI, ≥ 99.5% purity), hydrogen peroxide (H_2_O_2_, 30% *w*/*w*), H_2_SO_4_ (98%), glyphosate (OH)_2_P(O)CH_2_NHCH_2_CO_2_H, and NaOH (≥ 97%) were obtained from Sigma-Aldrich (Darmstadt, Germany). All solutions were prepared by using Milli-Q water (Elix-Milli Q Academic system (Millipore-Merck, Vimodrone, Italy).

### 3.2. Experimental Procedure

All experiments were performed at room temperature (20–22 °C), and the initial pH values were adjusted using diluted solutions of sulfuric acid (see below for the choice of H_2_SO_4_). In a typical experiment, 1 L of ultra-pure water containing 2 mg/L GLY was placed in a 1.2 L beaker. The value of the pH was monitored with a Checker pH meter (Hanna Instruments, Woonsocket, RI, US), and it was adjusted whenever required using diluted H_2_SO_4_. The required amounts of directly weighed (or pipetted) nZVI and H_2_O_2_ were added to already-prepared 2 mg/L GLY, and the whole system was then magnetically stirred for 1 h of reaction time. (Longer times would be uninteresting within the framework of water treatment.) After 1 h, a few mL of the sample was withdrawn, filtered (nylon 0.45 μm, VWR, Radnor, PE, USA), and injected into an ion chromatograph (Thermofisher-Dionex DX-100, Sunnyvale, CA, USA) equipped with a 200 μL loop, using 17 mM NaOH as the eluent. GLY was separated and detected with an IonPac AS16 column (250 × 4 mm, Thermofisher-Dionex, Sunnyvale, CA, USA), a Dionex ASRS 4 mm membrane suppressor, and a conductivity detector. The same experimental procedure was adopted to check for the adsorption efficiency of GLY using nZVI, with the exception that H_2_O_2_ was not added in this case. All experiments were conducted in triplicate.

The concentration of dissolved iron leached during the nZVI–Fenton treatment was determined via inductively coupled plasma–optical emission spectroscopy (ICP–OES, Agilent 5100, Santa Clara, CA, USA). Emission of atomic Fe was quantified at 238.204 nm, and the Fe-detection limit with this technique was at the level of μg/L. By comparison, the limit for Fe in wastewater is 4 mg/L according to Italian law [Legislative decree 152/06].

In tap-water experiments, tap water (from Turin, Italy) was spiked with GLY to achieve the desired 2 mg/L concentration. Then, the same procedure (as described earlier) was followed, but conductivity was also monitored throughout the reaction. Common inorganic anions in tap water were determined via suppressed anion chromatography.

Before deciding how to adjust the pH value, preliminary experiments were performed to minimize chromatographic interferences of acids (HClO_4_, HCl, and H_2_SO_4_) and buffers (phosphate and acetate) in the detection of GLY. Chromatographic interference was observed between GLY and HClO_4_, which was thus excluded. Although chloride did not interfere with GLY detection, HCl was not chosen because of the ability of chloride ions to scavenge HO^•^ in acidic solutions [48,54]. Phosphate buffer was not selected, owing to the very close elution of phosphate and GLY, which is due to the similar selectivity usually exhibited by chromatographic columns for these anions. Acetate buffer was tested but excluded due to ineffectiveness. (The orange-red Fe^2+^-acetate complex is possibly unreactive within the framework of nZVI + H_2_O_2_.) Therefore, H_2_SO_4_ was finally selected to adjust the pH value, with consideration of the high chromatographic resolution between sulfate and GLY ions.

## 4. Conclusions

Significant GLY degradation was obtained in the pH range of 3–4 using the nZVI–Fenton technique after optimization, which encompassed a variety of conditions of nZVI loading and H_2_O_2_ concentration. Unfortunately, the decrease in Fenton reactivity with an increase in pH prevented effective degradation to be achieved at either pH 5 or 6. GLY removal was also obtained at pH 3 with nZVI alone, without H_2_O_2_, but the required loading (300 mg/L nZVI) was 15–30 times higher than the loading required in nZVI–Fenton. That issue would be reflected in rather high costs for water treatment with nZVI alone.

In the case of tap water, complete (100%) GLY removal was achieved at pH 4 with 20 mg/L nZVI and 150 + 50 μM H_2_O_2_ (i.e., 150 μM added at t = 0, and 50 μM added after 30 min reaction time). Overall, water treatment costs for GLY with nZVI + H_2_O_2_ at pH 4 would be very reasonable in terms of the expenditures for chemical reagents (a total of USD 0.07/m^3^, of which approximately 30% would be for nZVI + H_2_O_2_, and the rest would go to pH-adjustment reagents). Interestingly, these same conditions would also allow for the removal of additional emerging contaminants (pharmaceuticals) from the water matrix [29,42].

Potential drawbacks of water treatment in acidic conditions are the elevated costs for pH adjustment, the increase in water conductivity following the acidification necessary for the Fenton process to work, the basification at the end of treatment to allow for water discharge, as well as the leaching of iron from the nZVI due to increased iron solubility at an acidic pH. As far as pH adjustment costs are concerned, we show here that they make up ~70% of the total reagent costs in the case of treatment at pH 4, and even ~80% for treatment at pH 3. With these limitations in mind, in this work, nZVI–Fenton was also tested at pH values of 5 and 6 but, unfortunately, no GLY degradation was achieved under these conditions. Water treatment at pH 4 would be quite satisfactory, however, because overall reagent costs would still be low, and the increase in conductivity was quite small, as compared, for instance, to treatment at pH 3. (Tap-water conductivity more than doubled upon treatment at pH 3, while a 40% increase is expected to occur at pH 4). Therefore, final water conductivity would still allow for water reuse in agriculture, which is a very demanding field as far as conductivity requirements are concerned. Furthermore, iron leaching from nZVI at pH 4 was low enough for treated water to meet the legal limits for safe discharge into the environment, while the same would not occur in the case of treatment at pH 3.

## Figures and Tables

**Table 1:** Concentration values of different anions in tap water.

Parameter	Measured Value (mg/L)
Chloride (Cl^−^)	26
Sulfate (SO_4_^2−^)	44
Nitrate (NO_3_^−^)	16
Nitrite (NO_2_^−^)	<0.05

## Data Availability

Not applicable.

## Sample Availability

Samples of the used reagents are available from the authors.
